# Supplementary material for: Ocean internal tides suppress tropical cyclones in the South China Sea
Source: Nat Commun. 2024 May 9;15:3903. doi: 10.1038/s41467-024-48003-y (PMC11082210; doi:10.1038/s41467-024-48003-y)
Supplement: Supplementary file 1 — Supplementary Information [file 41467_2024_48003_MOESM1_ESM.pdf]

Supplementary Information for

Ocean Internal Tides Suppress Tropical Cyclones in the South China Sea

Shoude Guan, Fei-Fei Jin*, Jiwei Tian*, I-I Lin*, Iam-Fei Pun, Wei Zhao, John Huthnance, Zhao Xu, Wenju Cai, Zhao Jing, Lei Zhou, Ping Liu, Yihan Zhang, Zhiwei Zhang, Chun Zhou, Qingxuan Yang, Xiaodong Huang,Yijun Hou, & Jinbao Song

*Corresponding authors. Email: jff@hawaii.edu, tianjw@ouc.edu.cn, or iilin@ntu.edu.tw

The supplementary information includes:

Figs. S1 to S13

Tables. S1 and S2

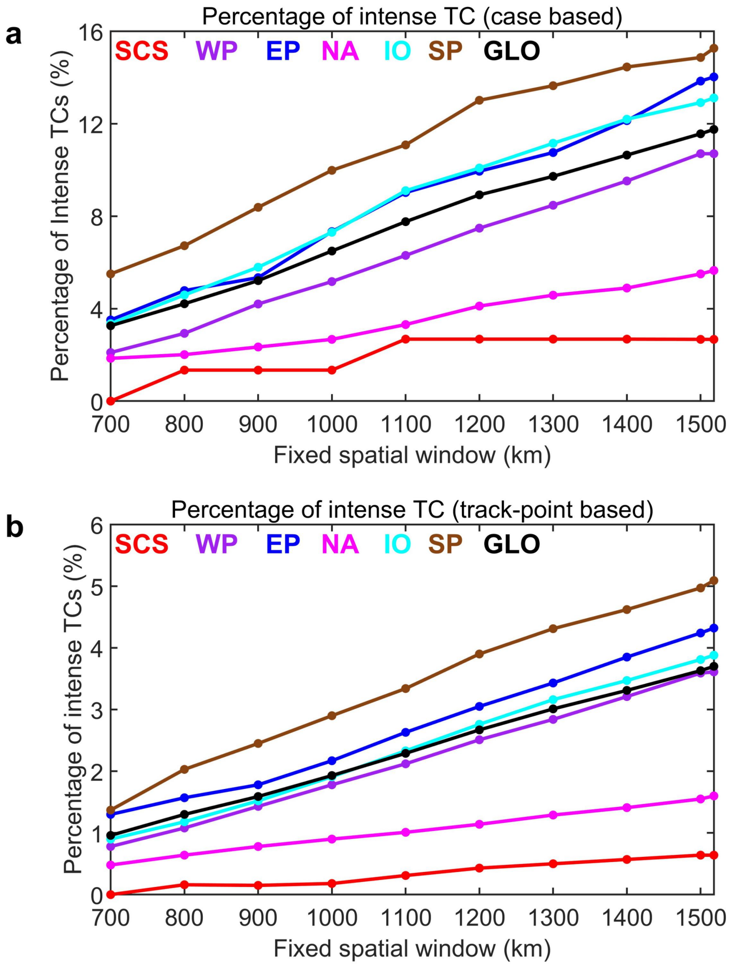

Fig. S1 | Percentage of intense tropical cyclones (TCs) based on the fixed-window analysis. The analysis compares the percentage of intense TCs, calculated for TCs in other TC-active oceans travelling the same distance as those in the South China Sea (SCS)**. a** Result from the TC-case based method. **b** Result from the TC track-point based. To avoid interference, TCs generated in the western North Pacific and translated into the SCS are first pre-excluded, i.e., only TCs locally generated in the SCS are considered for the SCS analysis. For TCs of the other five TC-active ocean basins, we only account for the segment from genesis to a traveling distance from 700 to 1518 km for each TC. TC-active oceans include the western North Pacific Ocean (WP), the South China Sea (SCS), the North Atlantic Ocean (NA), the eastern North Pacific Ocean (EP), the Indian Ocean (IO), and the South Pacific Ocean (SP). GLO means the global average over the five TC-active basins except the SCS. The SCS percentage of intense TCs is always the lowest. Thus, the relatively small basin size cannot explain the lowest percentage of intense TCs in the SCS. Source data are provided with this paper.

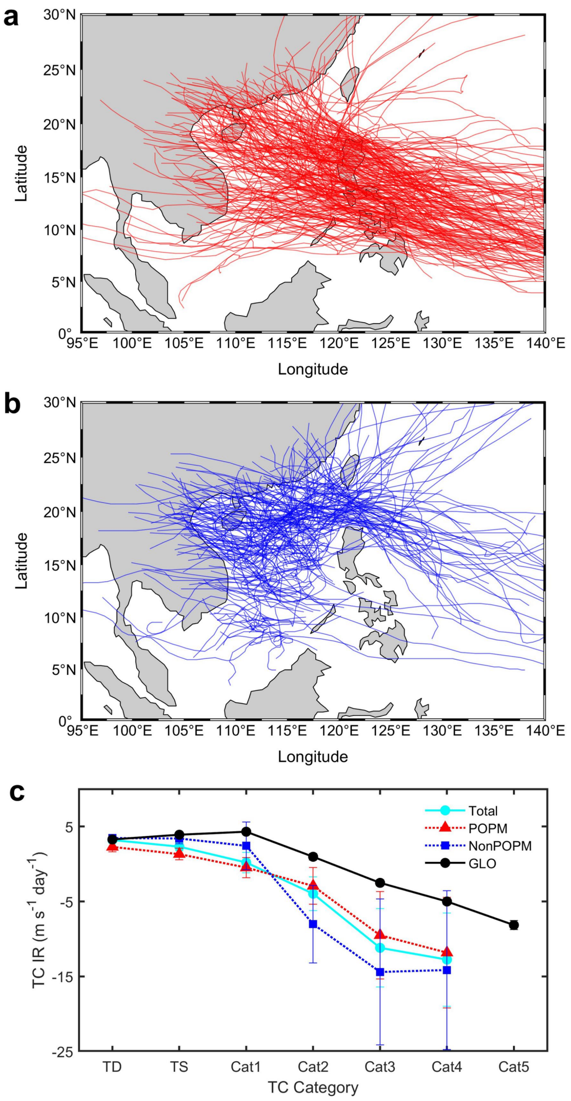

Fig. S2 | Tracks and intensification rates (IRs) for tropical cyclones (TCs) that enter the South China Sea (SCS) passing over the Philippine mountains (POPM) or not (NonPOPM). **a** Tracks of POPM subset (236 TCs); **b** Tracks of NonPOPM subset (209 TCs); **c** Average IRs versus TC category for all SCS TCs (Total, cyan), POPM subset (red), and NonPOPM (blue) subset, as well as the average IR in other five TC-rich oceans (GLO, black). To increase the sampling number, all TC track points not within 100 km of land are used here to calculate IRs. Error bars indicate the 90% confidence interval. Source data are provided with this paper.

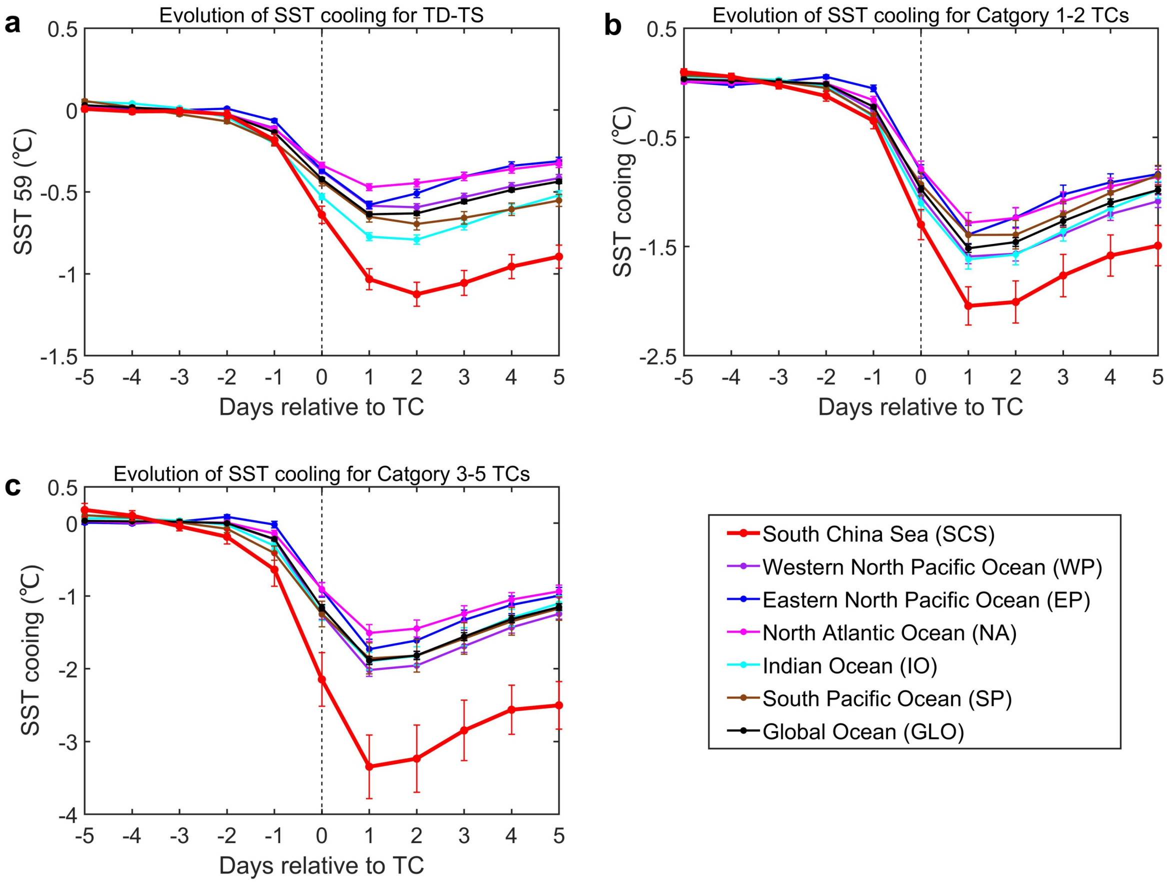

Fig. S3 | Composites of sea surface temperature (SST) cooling evolution before, during, and after tropical cyclone (TC) passage. a For tropical depression (TD) and tropical storm (TS). **b** For Category 1–2 TCs. **c** For Category 3–5 TCs. The vertical dashed lines indicate the time of TC passage. Error bars indicate the 90% confidence interval. During and after TC passage, cooling effect in the SCS is always the strongest among global TC-active oceans. Source data are provided with this paper.

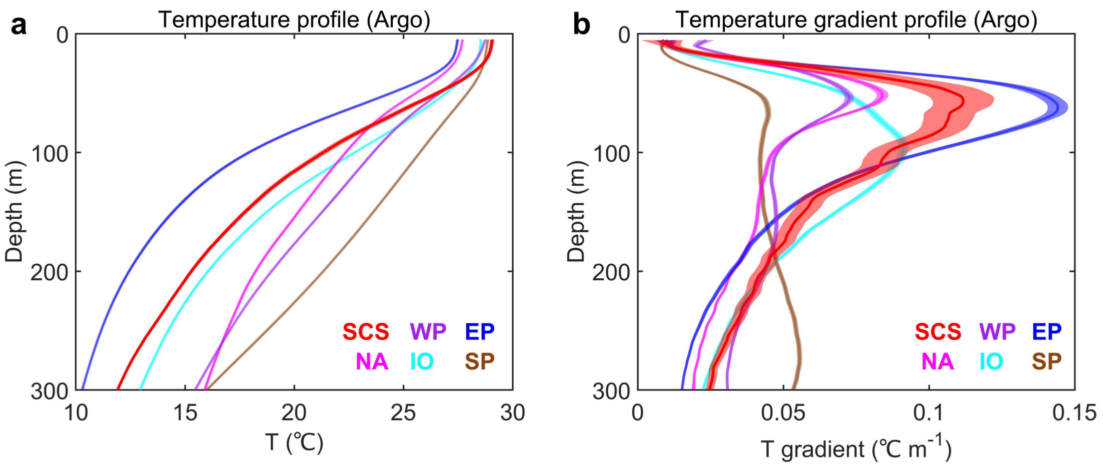

Fig. S4 | Upper ocean thermal stratification of tropical cyclone-active (TC-active) oceans in the TC season. a Area-averaged climatological temperature profile (T) derived from Argo data in each region during 2002–2019 in TC season. **b** As in (**a**), but for area-averaged climatological vertical temperature gradient ($\frac{\partial T}{\partial z}$). The shading indicates the 99% confidence interval. TC-active oceans include the South China Sea (SCS), the Western North Pacific Ocean (WP), the North Atlantic Ocean (NA), the Eastern North Pacific Ocean (EP), the Indian Ocean (IO), and the South Pacific Ocean (SP). Although the stratification in the SCS is strong, the stratification in the EP is even stronger, favoring stronger sea surface temperature (SST) cooling in the EP than that in the SCS under the same TC attributes. Source data are provided with this paper.

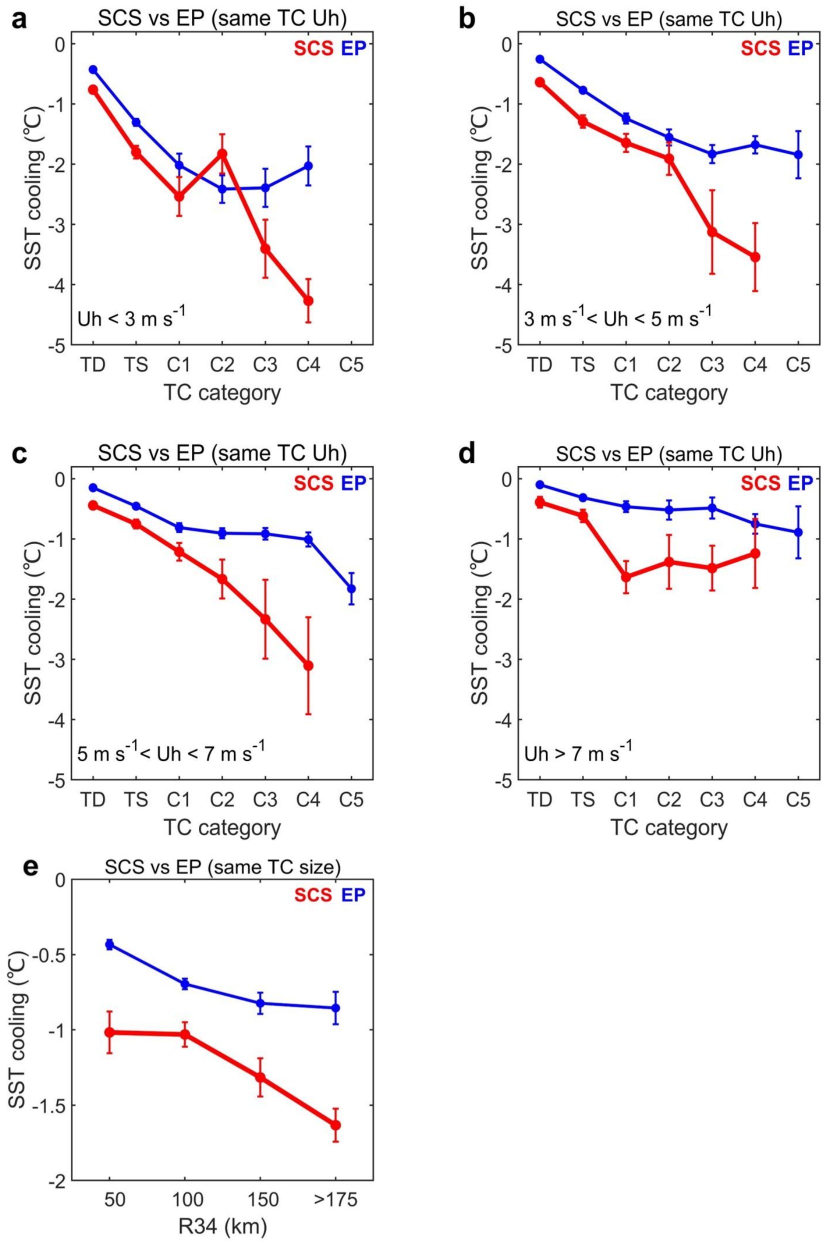

Fig. S5 | Tropical cyclone-induced (TC-induced) sea surface temperature (SST) cooling in the South China Sea (SCS) and Eastern Pacific (EP) under the same TC attributes. a Under the translation speed (Uh) ranges of Uh < 3 m s^{-1}. **b** Under Uh ranges of 3 m s^{-1} < Uh < 5 m s^{-1}. **c** Under Uh ranges of 5 m s^{-1} < Uh < 7 m s^{-1}. **d** Under Uh ranges of Uh > 7 m s^{-1}. **e** Under the same TC size (represented by the radius of 34 kts wind, R34). Error bars indicate the 90% confidence interval. Despite the weaker stratification, the SCS cooling is larger than that in the EP at the same TC intensity and translation speed. Source data are provided with this paper.

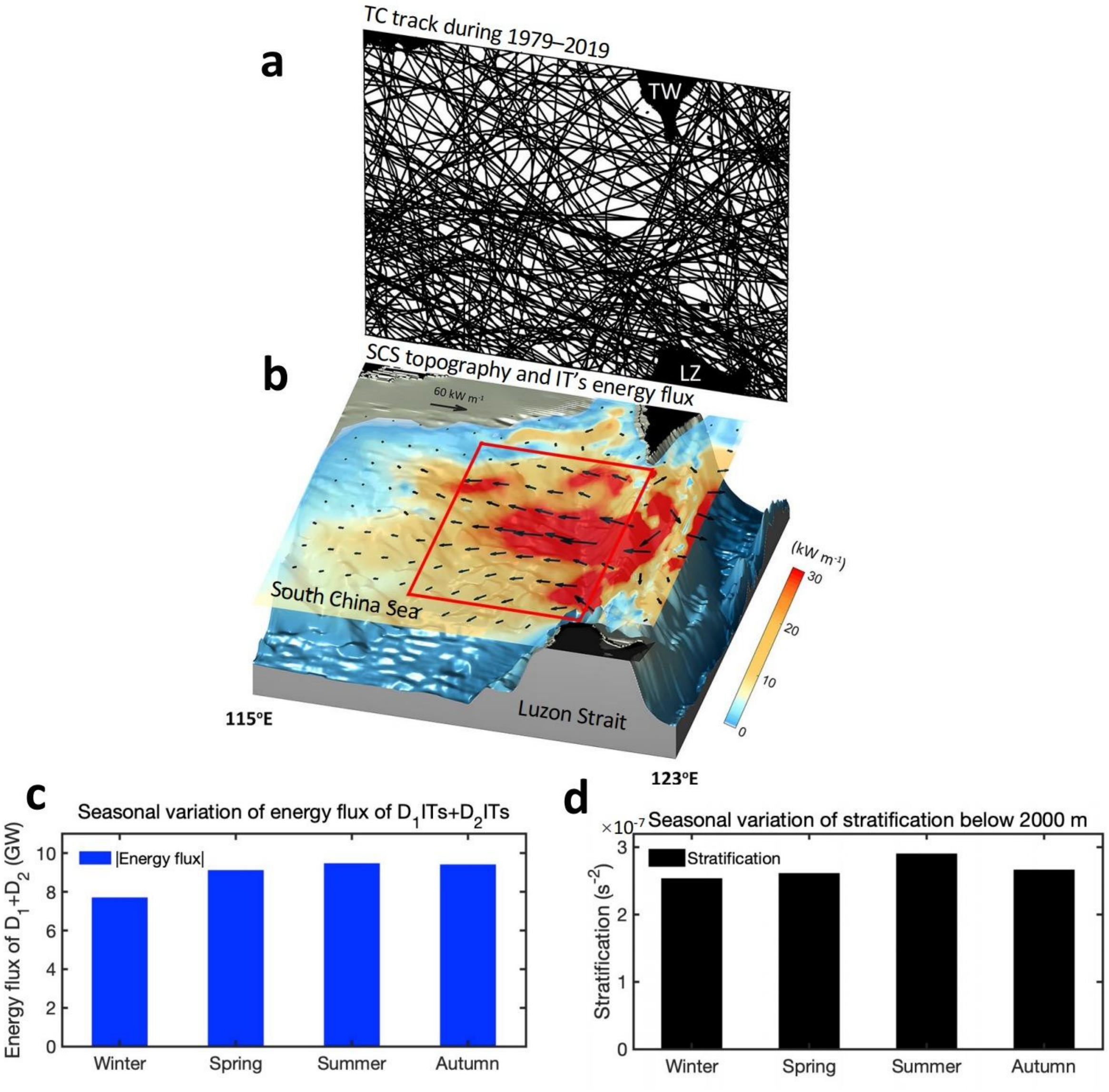

Fig. S6 | Schematic diagram illustrating tropical cyclones (TCs) and background internal tides (ITs) in the South China Sea (SCS). a TCs passing over the SCS during 1979–2019. **b** Topography (3D view; background color) and MITgcm model-simulated total energy flux (arrows) of diurnal and semidiurnal ITs in the SCS. The red box represents the chosen region for the calculation of seasonal variation. **c** Seasonal variation of stratification averaged below 2000 m. **d** Seasonal variation of region-integrated internal tidal energy flux at the Luzon Strait. Stratification and energy flux of diurnal and semidiurnal ITs are derived from the MITgcm LLC4320 simulation (with a horizontal resolution of 1/48° with 90 vertical layers over the globe, for 14 months from Sep. 2011 to Nov. 2012) by the ECCO team (Data source: ECCO Data Portal (https://data.nas.nasa.gov/ecco/data.php). ITs in the SCS, which are the most powerful in global oceans, are located *en route* of the frequent TC passages. Source data are provided with this paper.

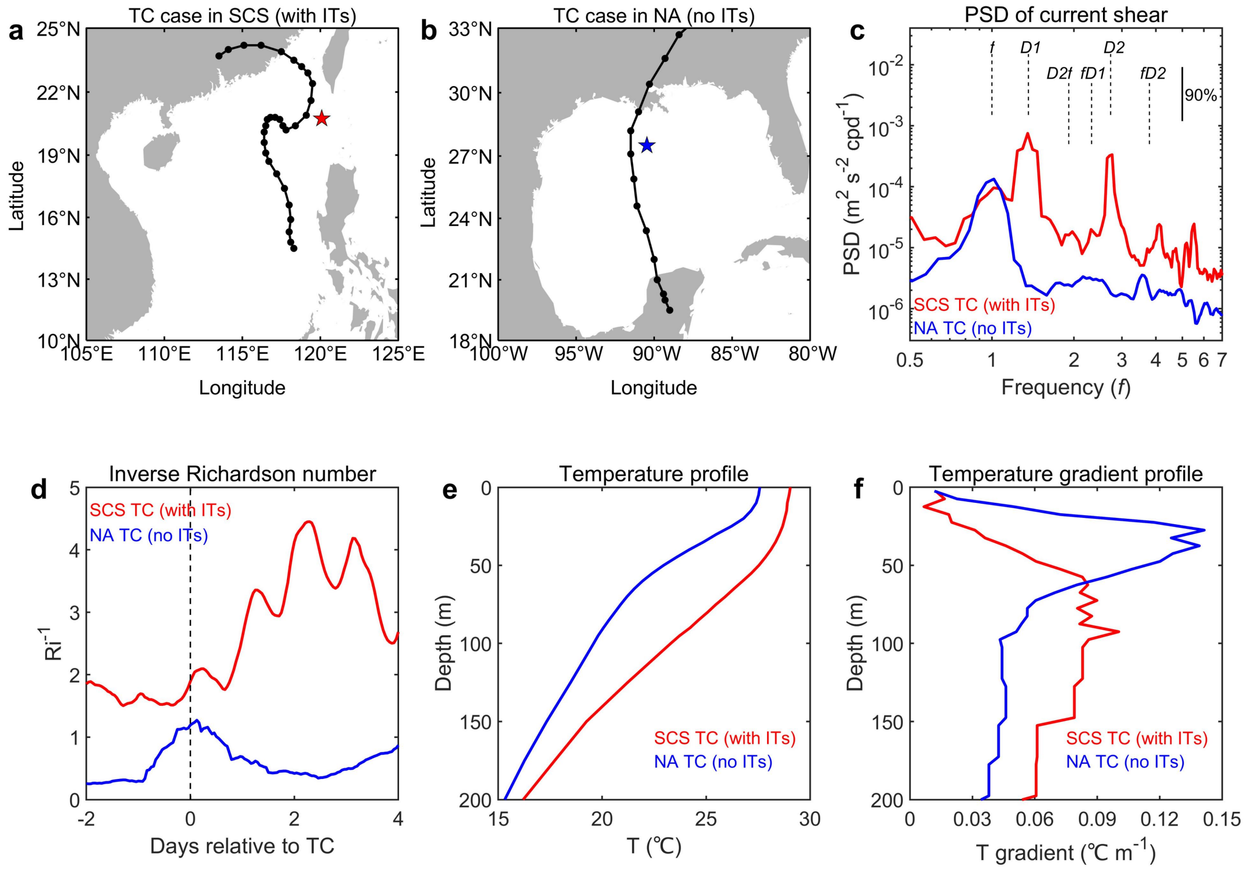

Fig. S7 | Comparison of dynamical response to tropical cyclones (TCs) between the South China Sea (SCS) and the North Atlantic (NA). a Track (black dot line) of TC Lionrock (2010) and the mooring position (red star; 20.5°N, 120.2°E) in the SCS, where strong background internal tides (ITs) are present. **b** Track (black dot line) of TC Bill (2003) and the buoy position (blue star; 27.5°N, 90.5°W) in the NA, where very few ITs are present. **c** As Fig. 4b in the main text, but for the observed current shear spectrum. **d** Mooring/buoy-observed, upper 150 m average inverse Richardson numbers (Ri^{-1}) during TC passage with (red; SCS) and without (blue; NA) background ITs. **e** Pre-TC ocean temperature profiles at the mooring (buoy) position in the SCS (NA) in the month of the TC passage. **f** As in (**e**), but for vertical temperature gradient profiles. Under similar TC conditions and even somewhat weaker subsurface thermal stratification than NA case, the *in-situ* observations indicate much stronger sea surface temperature (SST) cooling (Fig. 4d) in the SCS, due to the enhanced subsurface shear instability and entrainment by background ITs. Source data are provided with this paper.

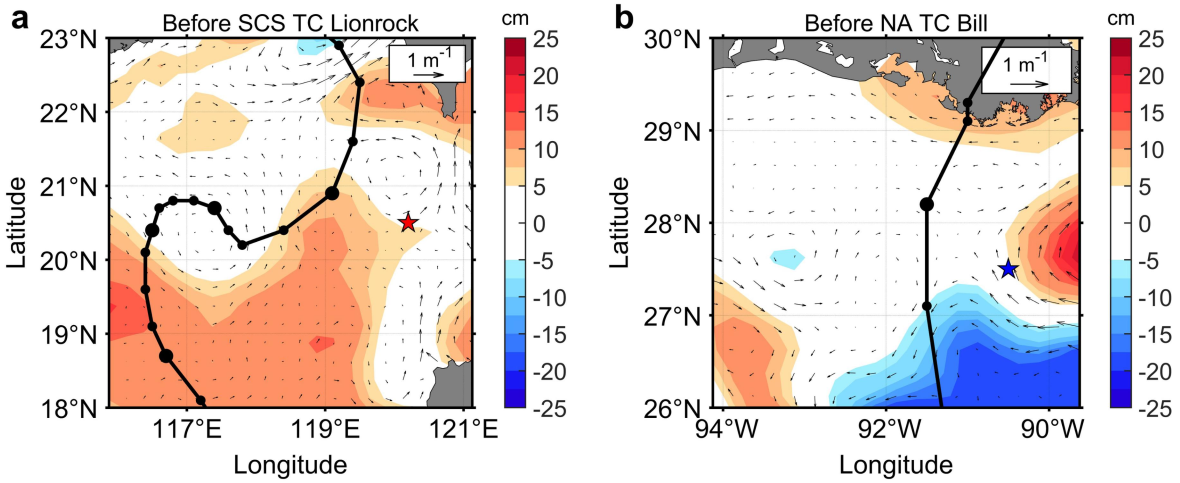

Fig. S8 | Sea level anomaly and geostrophic currents before the passage of tropical cyclones (TCs) in the South China Sea (SCS) and the North Atlantic (NA). a Sea level anomaly (color) and geostrophic current (black arrow) fields before TC Lionrock (2010) passing the mooring (red star) in the SCS. **b** Sea level anomaly (color) and geostrophic current (black arrow) fields before TC Bill (2003) passing the buoy (blue star) in the NA. Black solid lines and dots denote the tracks of TC Lionrock and Bill. The sea surface anomaly and geostrophic currents are obtained from the Copernicus Marine Environment Monitoring Service. Source data are provided with this paper.

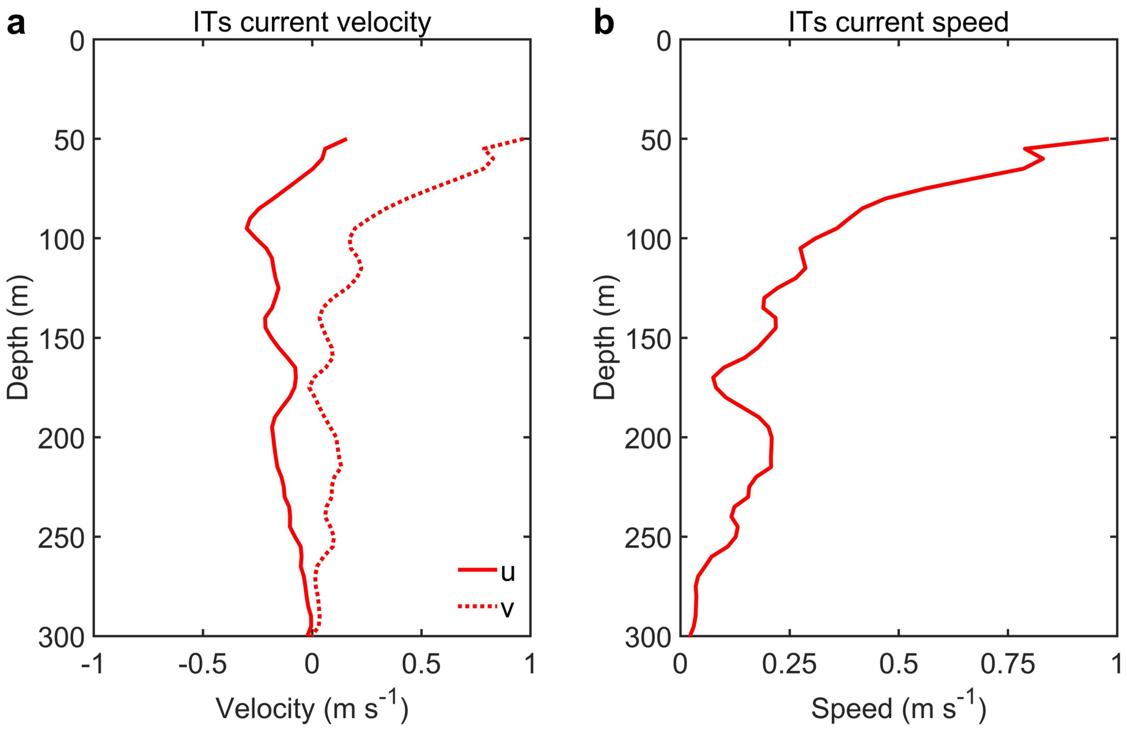

Fig. S9 | Current profile of internal tides (ITs) used in the Price model experiments. a Current's east-west component (*u*) and north-south component (*v*). **b** Absolute current speed. These profiles were observed from mooring R1 in ref.[28] (Fig. S10a). Source data are provided with this paper.

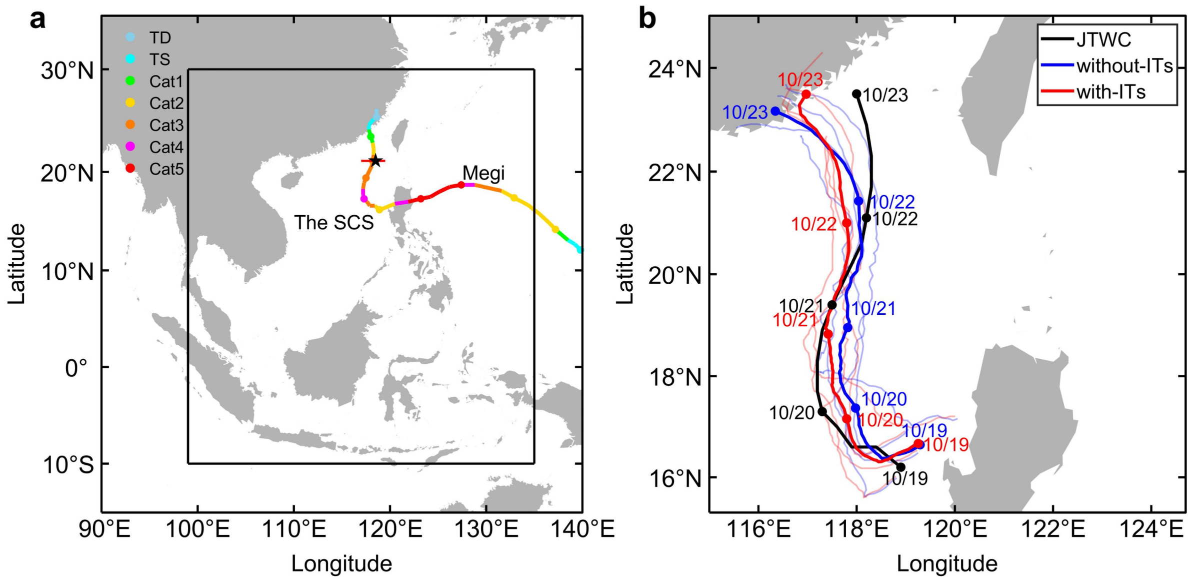

Fig. S10 | Tracks of tropical cyclone (TC) Megi in ROMS and WRF model experiments. a Best track of TC Megi (October, 2010) in the ROMS model experiments. Different colors of the curve and points stand for different TC intensities according to the Saffir-Simpson scale. The squared box with solid lines represents the model domain. The black star indicates the mooring R1 in ref.[28]. The 21°N section analyzed in Fig. 5c and 5d is indicated by the horizontal red line. **b** The JTWC and WRF-simulated tracks of Megi (2010). Black, JTWC observation; thick blue, ensemble mean track of four without-ITs runs; thick red, ensemble mean track of four with-ITs runs. The time is indicated with numbers near TC tracks. The thin-blue (red) lines are the four simulated tracks of the without-ITs (with-ITs) ensemble experiment. Source data are provided with this paper.

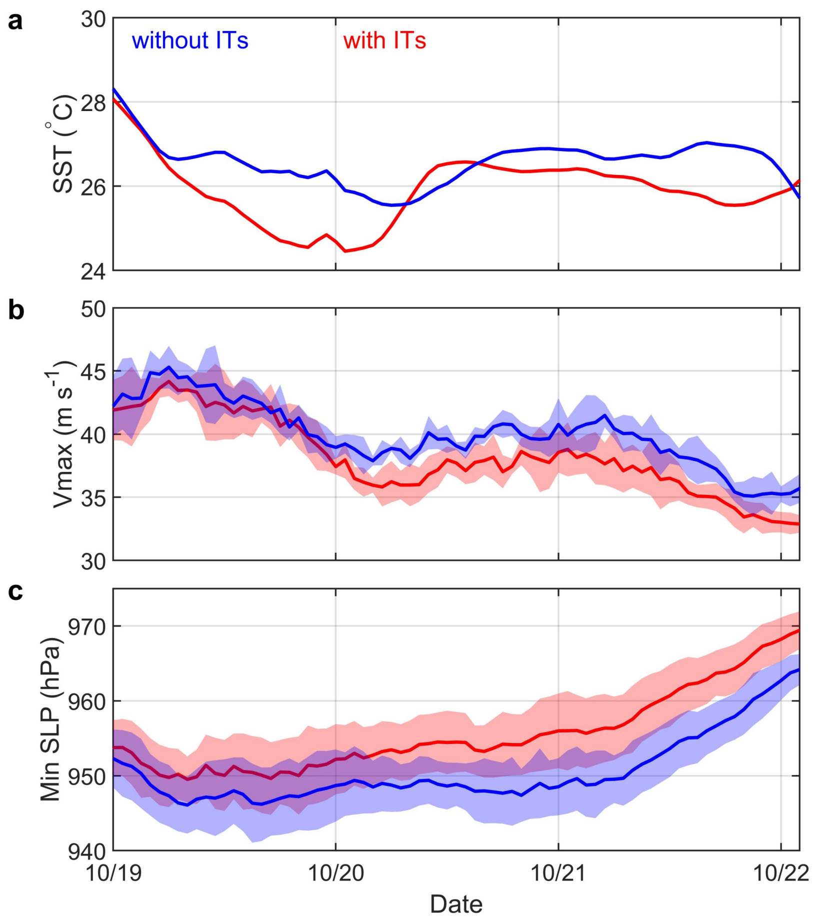

Fig. S11 | Simulated sea surface temperature (SST) cooling and intensity of tropical cyclone (TC). a Along-TC-track SST simulated by ROMS experiments. **b** Maximum 10 m wind speed (Vmax). **c** Minimum sea level pressure (Min SLP). The blue and red lines are ensemble means of without-ITs and with-ITs experiments, respectively. The shadings are the standard deviations of the ensemble members for each experiment. The along-track SST is defined as the spatially averaged SST within 100 km of the TC center. Source data are provided with this paper.

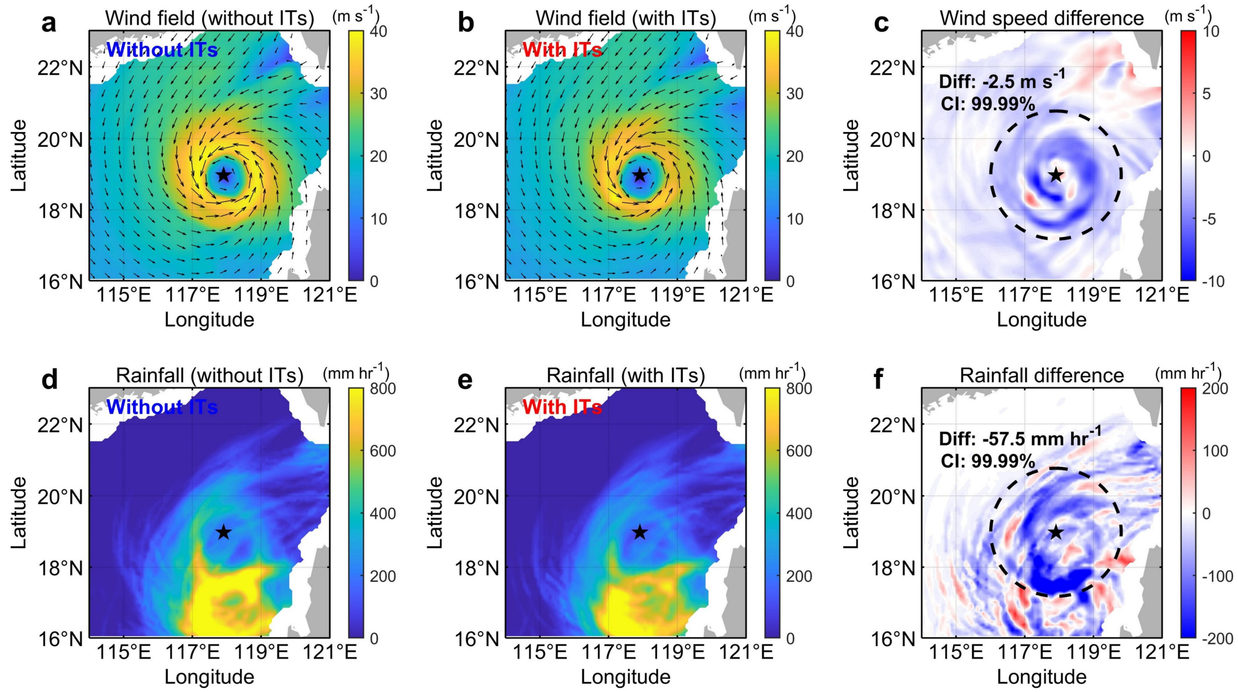

Fig. S12 | Simulated wind speed and rainfall associated with tropical cyclone (TC) snapshots at 20 UTC 20 Oct. a Wind speed (m s^{-1}) in the without-ITs simulation. The background color indicates the magnitude of wind speed and arrows indicate wind direction. Data within 50 km from the land have been removed. **b** As in (**a**), but for the with-ITs simulation. **c** Difference of wind speed (with-ITs simulation minus without-ITs). **d** Rain rate (mm hr^{-1}) in the without-ITs simulation. **e** As in (**d**), but for the with-ITs simulation. **f** Difference of rain rate (with-ITs simulation minus without-ITs). The black star represents the TC center and the dashed-black circle represents 200 km radii from the TC center. The average value of difference within 200 km of the TC center and the confidence level are labeled on the upper-left corner. In the with-ITs simulation, TC wind speed and rainfall are both suppressed, and are weaker than those from without-ITs simulation at the 99% significance level. Source data are provided with this paper.

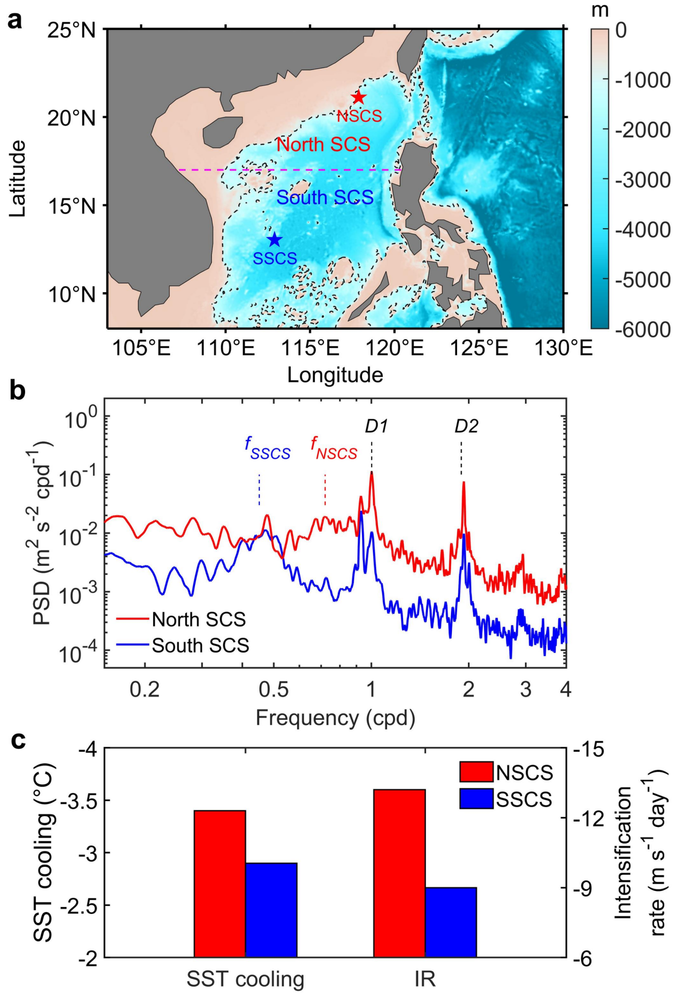

Fig. S13 | Comparison of internal tide (IT) effect in the north and south part of the South China Sea (SCS). a Locations of the two moorings in the north and south SCS. Mooring NSCS (red star; 21.1°N, 117.9°E) is located in the north SCS (north of 17°N). Mooring SSCS (blue star; 13.0°N, 112.9°E) is located in the south SCS (south of 17°N). The background color represents the topography of the SCS. Black dashed contours represent the 1000 m isobath. The boundary (17°N) of south and north SCS is indicated by the magenta dashed line. **b** Average power spectrum of the horizontal currents of internal waves in the north (red) and south (blue) SCS. The unit of x-axis is cpd (cycles per day). The frequencies of NIW (f), background diurnal (D1), and semidiurnal (D2) ITs are indicated by vertical dashed lines. The power spectra are derived from synchronous ocean current observations at the two moorings from July 2016 to July 2017. **c** TC-induced sea surface temperature (SST) cooling and TC intensification rate for intense TCs in the north and south SCS, derived from composite results of satellite observations. Source data are provided with this paper.

Table S1 | Comparison of tropical cyclone (TC) attributes among the six TC-active oceans.

Oceans		SCS	WP	EP	NA	IO	SP	GLO
TC Frequency (Counts/unit area)		37.8	25.9	24.0	8.9	12.8	9.3	14.5
Percentage of Intense TCs (%, TC track-point based)		3.2	17.4	9.5	8.5	8.0	7.8	11.1
Percentage of Intense TCs (%, TC case based)		8.6	33.8	24.0	13.6	22.9	21.1	24.1
Probability of rapid intensification (%, TC-track-point based)		2.4	8.2	6.1	5.3	5.5	7.1	6.5
Probability of rapid intensification (%, TC case based)		10.4	38.7	33.2	26.4	29.4	35.3	32.7
Climatological sea surface temperature (SST, °C)		28.8	28.6	26.9	27.7	28.1	28.5	27.9
Climatological Potential Intensity ($m\ s^{-1}$)		71.4	71.2	61.3	68.6	71.4	77.4	70.3
Climatological Relative Humidity (%)		65.1	54.7	44.4	44.9	40.0	48.1	45.8
Average Vmax ($m\ s^{-1}$)		23.0±0.4	30.1±0.2	26.2±0.2	25.9±0.3	25.0±0.2	24.8±0.3	26.9±0.1
Intensification Rate ($m\ s^{-1}\ day^{-1}$)	TD–C5	1.4±0.4	3.9±0.2	1.6±0.2	2.1±0.2	2.3±0.1	3.6±0.3	2.6±0.1
	C3–C5	−11.7±5.3	−3.7±0.4	−6.4±0.5	−2.2±0.7	−5.1±0.7	−3.9±1.4	−4.5±0.3
Vertical Wind Shear ($m\ s^{-1}$)	TD–C5	7.9±0.1	7.2±0.1	6.4±0.1	9.4±0.2	8.2±0.1	6.9±0.1	7.3±0.1
	C3–C5	5.9±0.7	5.8±0.1	4.5±0.1	5.8±0.3	6.1±0.3	5.5±0.4	5.5±0.1
Pre-TC SST (°C)	TD–C5	28.8±0.1	28.8±0.1	27.5±0.1	28.3±0.1	28.4±0.1	28.8±0.1	28.4±0.1
	C3–C5	28.9±0.2	28.8±0.1	27.8±0.1	28.8±0.1	28.3±0.1	28.8±0.1	28.5±0.1
SST Cooling (°C)	TD–C5	−1.3±0.1	−1.0±0.1	−0.8±0.1	−0.7±0.1	−1.0±0.1	−0.8±0.1	−0.9±0.1
	C3–C5	−3.1±0.3	−1.8±0.1	−1.6±0.1	−1.4±0.1	−1.8±0.1	−1.7±0.1	−1.7±0.1
Heat Flux ($W\ m^{-2}$)	TD–C5	284.5±45.6	382.5±53.7	326.6±31.0	441.2±28.6	320.1±39.9	335.3±34.9	344.0±41.5
	C3–C5	364.9±213.7	526.8±142.2	573.4±107.2	687.3±98.3	548.5±134.7	524.3±132.5	540.5±133.0

*The "±" values indicate the 90% confidence interval. TC-active oceans include the western North Pacific Ocean (WP), the South China Sea (SCS), the North Atlantic Ocean (NA), the eastern North Pacific Ocean (EP), the Indian Ocean (IO), and the South Pacific Ocean (SP). GLO means the global average over the five TC-active basins except the SCS. For the probability of rapid intensification and percentage of intense TC estimation, two methods are employed to ensure robustness, for details see Methods.

Table S2 | WRF physics options used in the WRF sensitivity experiments.

Longwave Radiation Physics	RRTM scheme
Shortwave Radiation Physics	Dudhia
Surface Layer Model	Revised MM5 Monin-Obukhov scheme
Land Surface Model	Thermal diffusion scheme
Microphysics Scheme	WSM 6-class graupel scheme
PBL Scheme	MYNN 2.5 level TKE scheme
Cumulus Scheme	Kain-Fritsch (new Eta) scheme
